# Personal

**Published:** 1897-11

**Authors:** 


### Personal.

Dr. Ernest Wende, health commissioner of Buffalo, and Dr. F. C. Gram, registrar of vital statistics for the health department, attended the meeting of the American Public Health Association at Philadelphia, October 26-28, 1897, as official delegates from the Buffalo department of health.

Dr. Henry J. Mulford, of Buffalo, has moved his residence to the Markeen, corner of Main and Utica streets. His office remains at 466 Franklin street. His practice is limited to diseases of the nose, throat and ear. Hours, 10 A. M. to 12 M., 2 to 4 P. M. Sundays, 12 M. to 1 P. M.

Dr. Frank Whitehill Hinkel, of Buffalo, sailed for Europe early in October, expecting to spend several months in recreation and study. He will then return to his home and resume the practice of laryngology at his office, 305 Delaware avenue.

Dr. Stephen Smith, of New York, has been appointed by Mayor Strong a commissioner of charities to fill a vacancy. This is a most excellent appointment and will strengthen the board of charities in a marked degree.

Dr. William C. Krauss, of Buffalo, was elected president of the Medical Association of Central New York at its recent meeting held in this city. This is a fitting recognition of Dr. Krauss's ability and interest in the affairs of the association.

Dr. John E. Bacon, of Buffalo, announces his removal from 79 Niagara square to the Newport, 176 Prospect avenue, corner of Virginia. Telephone, Tupper 286. Hours, 8-10 A. M., 2-4 and 7-8 P. M.

Dr. Lorenzo Burrows, of Buffalo, has removed from 388 Franklin street to 482 Franklin street. His practice is limited to diseases of the eye and ear. Hours, 9 A. M. to 1 P. M.

